# Supplementary material for: Acute Stress and Autoimmune Markers: Evaluating the Psychoneuroimmunology Axis in Firefighter Recruits
Source: Int J Mol Sci. 2025 Apr 22;26(9):3945. doi: 10.3390/ijms26093945 (PMC12071583; doi:10.3390/ijms26093945)
Supplement: Supplementary file 1 [file ijms-26-03945-s001.zip › ijms-3527724-supplementary.pdf]

Supplemental Table S1. Demographics Data.

	Number	%
Gender		
Male	25	96.2
Female	1	3.8
Age		
19-29	17	65.4
30-39	8	30.8
40-49	1	3.8
Race		
White	26	100
Black	0	0
Asian	0	0
Pacific Islander	0	0
Native American	0	0
Ethnicity = Hispanic	2	7.7
Prior military experience	4	15.4
Prior EMT experience	12	46.2
Prior Dx of PTSD	1	3.8
Prior Dx of Autoimmune	0	0
Actively taking immunosuppressant or hypnotic	0	0
	Mean (Std. Dev.)	Range
Prior EMT Experience (years)	4.63 (4.47)	1-18

n=26

Supplemental Table S2. Life Events Checklist (LEC-5)

LEC-5 Standard

Instructions: Listed below are a number of difficult or stressful things that sometimes happen to people. For each event check one or more of the boxes to the right to indicate that: (a) it happened to you personally; (b) you witnessed it happen to someone else; (c) you learned about it happening to a close family member or close friend; (d) you were exposed to it as part of your job (for example, paramedic, police, military, or other first responder); (e) you're not sure if it fits; or (f) it doesn't apply to you.

Be sure to consider your *entire life* (growing up as well as adulthood) as you go through the list of events.

Event	Happened to me	Witnessed it	Learned about it	Part of my job	Not sure	Doesn't apply
1. Natural disaster (for example, flood, hurricane, tornado, earthquake)						
2. Fire or explosion						
3. Transportation accident (for example, car accident, boat accident, train wreck, plane crash)						
4. Serious accident at work, home, or during recreational activity						
5. Exposure to toxic substance (for example, dangerous chemicals, radiation)						
6. Physical assault (for example, being attacked, hit, slapped, kicked, beaten up)						
7. Assault with a weapon (for example, being shot, stabbed, threatened with a knife, gun, bomb)						
8. Sexual assault (rape, attempted rape, made to perform any type of sexual act through force or threat of harm)						
9. Other unwanted or uncomfortable sexual experience						
10. Combat or exposure to a war-zone (in the military or as a civilian)						
11. Captivity (for example, being kidnapped, abducted, held hostage, prisoner of war)						
12. Life-threatening illness or injury						
13. Severe human suffering						
14. Sudden violent death (for example, homicide, suicide)						
15. Sudden accidental death						
16. Serious injury, harm, or death you caused to someone else						
17. Any other very stressful event or experience						

Supplemental Table S3. Sensitivity/Specificity

Analyte	Minimum Detectable concentration (ng/mL)	Cross Reactivity
Cortisol	0.607	Negligible cross-reactivity between antibodies for an analyte and any of the other analytes
CRP	0.0022	Negligible cross-reactivity between antibodies for an analyte and any of the other analytes
PEDF	0.008	Negligible cross-reactivity between antibodies for an analyte and any of the other analytes
SAP	0.009	Negligible cross-reactivity between antibodies for an analyte and any of the other analytes
C4	0.0465	Negligible cross-reactivity between antibodies for an analyte and any of the other analytes

Supplemental Table S4. Descriptive Statistics Data

Time Point 1						
Variable	Mean	Std Dev	N	Median	Minimum	Maximum
Cortisol-A	733239.01	1053497.5	26	403184.36	8812.07	4310800
CRP-A	16396.43	33014.01	26	2711.88	29.49	148146.60
C4-A	8.46	5.88	26	6.78	1.76	24.09
PEDF-A	4.76	4.19	26	3.79	1E-08	17.66
SAP-A	12.44	15.02	26	7.69	1.42	77.85
Cortisol-B	393586.91	643750.71	26	110544.81	8469.01	2521400
CRP-B	3506.07	5809.19	26	781.51	9.49	23216.33
C4-B	8.24	6.27	26	5.62	1.06	20.58
PEDF-B	3.79	3.76	26	3.12	0.27	18.21
SAP-B	15.53	23.50	26	8.69	1.12	115.23

Time Point 2						
Variable	Mean	Std Dev	N	Median	Minimum	Maximum
Cortisol-A	5076960.74	13664247.8	26	1025710.84	13675.56	58854000
CRP-A	38577.53	86138.49	26	3684.81	11.74	345832.73
C4-A	12.16	10.25	26	8.00	1.76	44.78
PEDF-A	6.70	6.84	26	4.26	1.02	32.98
SAP-A	21.85	30.93	26	9.65	2.44	140.96
Cortisol-B	1779909.85	4214069.99	26	209396.69	2107.67	16772000
CRP-B	4503.79	7225.78	26	604.66	18.1	28345.35
C4-B	9.53	7.17	26	6.70	1.49	31.05
PEDF-B	5.12	5.83	26	3.22	0.81	25.33
SAP-B	30.89	61.25	26	9.17	1.49	39.51

Time Point 3						
Variable	Mean	Std Dev	N	Median	Minimum	Maximum
Cortisol-A	664874.55	903987.85	26	237417.99	2423.82	3158100
CRP-A	7099.72	14762.15	26	1061.67	9.34	72127.74
C4-A	11.33	15.52	26	5.74	1.69	65.07
PEDF-A	4.08	5.11	26	2.55	0.33	21.92
SAP-A	9.13	10.28	26	4.45	0.58	46.75
Cortisol-B	473151.87	788227.33	26	119424.11	18300.35	3238300
CRP-B	3078.58	10122.15	26	232.56	5.87	52953.79
C4-B	6.97	7.22	26	4.29	1.06	35
PEDF-B	2.63	3.44	26	1.41	0.26	15.71
SAP-B	8.06	9.17	26	4.34	0.63	39.51
